# Structure–dependent chlorine dioxide release from poly(acrylic acid) and hydroxyethyl cellulose hydrogels: Insights from real-time positron annihilation lifetime spectroscopy and quantum chemical calculations

**DOI:** 10.1016/j.fochx.2025.102782

**Published:** 2025-07-11

**Authors:** Pegah Poursafar, Adrienn Kazsoki, Barnabás Palcsó, Károly Süvegh, Romána Zelkó

**Affiliations:** a University Pharmacy Department of Pharmacy Administration, Faculty of Pharmaceutical Sciences, Semmelweis University, Hőgyes E. Street 7–9, H-1092 Budapest, Hungary; b Eötvös Loránd University Laboratory of Nuclear Chemistry, P.O. Box 32, H-1518 Budapest, Hungary

**Keywords:** Chlorine dioxide hydrogels, Controlled release, Positron annihilation lifetime spectroscopy, Quantum chemical modeling, Antimicrobial delivery, Chlorine dioxide (PubChem CID: 24870), Poly(acrylic acid) (PubChem CID: 6581), Hydroxyethyl cellulose (PubChem CID: 24846132), Acrylic acid (PubChem CID: 6581), Glucose (PubChem CID: 5793), Sodium hydroxide (PubChem CID: 14798), Water (PubChem CID: 962), Solvocid (PubChem CID: 24870), Hydroxyethyl (PubChem CID: 123), Carboxyl (PubChem CID: 23667)

## Abstract

Chlorine dioxide (ClO₂) is a potent disinfectant used in controlled-release systems for water treatment and antimicrobial purposes. This study investigates ClO₂ release kinetics in poly(acrylic acid) (PAA) and hydroxyethyl cellulose (HEC) hydrogels via positron annihilation lifetime spectroscopy and quantum chemical modeling. PAA hydrogels released ClO₂ 2.5 times faster than HEC, with a 68.7 % decrease in o-Ps lifetime intensity in 12 h, compared to 27.3 % for HEC. This rapid release is linked to PAA's linear, anionic structure, showing binding energies of −24.8 kJ/mol for ClO₂ and − 65.6 kJ/mol for water. HEC hydrogels showed concentration-dependent release; 4 %*w*/w HEC retained 57.6 % more ClO₂ after 24 h than 2 %w/w HEC. Quantum calculations confirmed stronger secondary bond energies for PAA (−24.8 kJ/mol) than HEC (−19.6 kJ/mol) with ClO₂, revealing structure-dependent release mechanisms. These results highlight polymer architecture's critical role in controlled-release behavior, guiding hydrogel-based antimicrobial delivery design for food preservation and water treatment.

## Highlights

• Poly(acrylic acid) releases ClO₂ 2.5-fold faster than cellulose hydrogels.
• Real-time PALS reveals polymer structure controls release kinetics.
• Quantum calculations explain the molecular basis of structure-dependent release.
• Higher polymer concentration enables sustained antimicrobial protection.
• Method enables rational design of tailored food preservation systems.

## 1 Introduction

Chlorine dioxide (ClO_2_) has emerged as one of the most potent and versatile disinfectants in modern antimicrobial applications, particularly valued for its superior efficacy in water treatment and food preservation system. In food preservation, ClO₂ is extensively used for fresh produce sanitization, achieving significant reductions in pathogenic microorganisms such as *E. coli* O157:H7, Salmonella, and *Listeria monocytogenes* on fruits and vegetables while extending shelf life and maintaining nutritional quality (Simonič, 2019; Andrés et al., 2022). Food processing facilities utilize ClO₂ for equipment disinfection, surface sanitization, and water treatment systems, where its broad-spectrum antimicrobial activity and minimal residue formation make it ideal for maintaining food safety standards (Petterson & Stenström, 2015). In water treatment applications, ClO₂ serves as a superior alternative to traditional chlorine-based disinfectants for drinking water disinfection, wastewater treatment, and industrial water systems, maintaining antimicrobial activity across a wide pH range while producing fewer harmful disinfection byproducts (Deborde & von Gunten, 2008). Unlike conventional chlorine-based disinfectants, ClO_2_ maintains its antimicrobial activity across a wide pH range and produces fewer harmful byproducts, making it an attractive option for both industrial and consumer applications (Andrés et al., 2022; Petterson & Stenström, 2015). The growing global challenges of foodborne pathogens, waterborne diseases, and antimicrobial resistance have intensified interest in developing controlled-release systems that can provide sustained antimicrobial activity while minimizing direct toxicity.

The integration of ClO_2_ into polymeric delivery systems, particularly hydrogels, represents a significant advancement in controlled-release technology for both food preservation and water treatment applications. For food preservation, hydrogel-based systems offer unique advantages as active packaging materials that can provide sustained antimicrobial protection while maintaining food quality and extending shelf life (Li & Mooney, 2016; Palcsó et al., 2019). In water treatment, hydrogel carriers enable controlled release of ClO₂ over extended periods, reducing the frequency of dosing and ensuring consistent disinfection efficacy (Seliktar, 2012). Hydrogels offer unique advantages as carriers for antimicrobial agents due to their ability to provide spatial and temporal control over drug release while maintaining biocompatibility and protecting labile compounds from degradation (Li & Mooney, 2016; Seliktar, 2012). The three-dimensional polymer network structure of hydrogels can be precisely tuned to achieve desired release kinetics through modification of crosslinking density, polymer composition, and architectural features, making them particularly suitable for applications requiring sustained antimicrobial activity (Peppas et al., 2006).

Among the various polymers suitable for hydrogel formation, poly(acrylic acid) (PAA) and hydroxyethyl cellulose (HEC) have attracted considerable attention for food and water applications due to their distinct molecular architectures, biocompatibility, and physicochemical properties. PAA is a linear synthetic polymer with ionizable carboxyl functional groups, exhibits excellent water retention and rapid response to environmental changes, making it suitable for applications requiring immediate antimicrobial action such as emergency water treatment or acute food safety interventions (Abdel-Halim, 2012; Terao et al., 2014). This polymer has found extensive applications in drug delivery, tissue engineering, and controlled-release systems, with its properties being well-documented for various therapeutic applications (Bardajee et al., 2012). Recent studies have demonstrated the potential of naturally derived multifunctional hydrogels in biomedical applications, such as diabetic wound healing, underscoring the versatility of hydrogel-based systems in addressing complex therapeutic challenges (Naeem et al., 2023; Shi et al., 2023). HEC, a naturally derived polymer with exceptional water-binding capacity and excellent biocompatibility, is particularly suitable for food-contact applications and long-term water treatment systems where sustained release and minimal toxicity are crucial (Arai & Shikata, 2020; Arfin & Bohidar, 2012). The presence of numerous hydroxyl groups along the polymer chain enables extensive hydrogen bonding interactions, contributing to controlled release behavior essential for maintaining consistent antimicrobial efficacy over extended periods (Liu et al., 2012).

Despite the importance of polymer architecture in controlling release behavior, the fundamental molecular-level mechanisms governing ClO_2_ interactions with different hydrogel systems remain poorly understood. The release kinetics of antimicrobial agents from polymer networks are influenced by complex physicochemical interactions, including diffusion processes, polymer-drug binding, matrix degradation, and swelling behavior (Ritger & Peppas, 1987; Siepmann & Peppas, 2012). Traditional analytical approaches often provide limited insight into these molecular-level interactions, necessitating the application of advanced characterization techniques that can probe polymer microstructures and dynamics in real-time (Hoare & Kohane, 2008). Positron annihilation lifetime spectroscopy (PALS) has emerged as a unique and powerful technique for investigating free volume characteristics and molecular dynamics in polymer systems (Klym et al., 2021). This non-destructive method provides quantitative information about cavity sizes and distributions within polymer networks, which are directly related to transport properties and release behavior (Dull et al., 2001). PALS is particularly valuable for studying hydrogel systems as it can monitor structural changes during swelling and drug release processes without altering the inherent properties of the materials. The technique's ability to probe cavities ranging from sub-nanometer to several nanometers makes it ideally suited for understanding how ClO_2_ molecules interact with and move through polymer networks (Bamford et al., 2003).

To complement experimental observations, quantum chemical calculations provide molecular-level insights into the binding interactions between ClO_2_, water, and polymer chains (Jones, 2015). These computational approaches can quantify the strength of secondary interactions, predict preferred binding configurations, and rationalize experimental release profiles based on fundamental thermodynamic principles (Burke et al., 2005; Perdew et al., 1996). The integration of experimental and computational methods offers a comprehensive understanding of structure-property relationships in controlled-release systems.

The present investigation aims to elucidate the fundamental mechanisms governing ClO_2_ release from PAA and HEC hydrogels through a combined experimental and computational approach. By correlating real-time PALS measurements with quantum chemical calculations, we seek to establish clear relationships between polymer architecture, molecular interactions, and release kinetics. This understanding is crucial for the rational design of next-generation antimicrobial delivery systems with tailored release profiles for specific applications in food preservation and water treatment.

## 2 Materials and methods

### 2.1 Materials

PAA with an apparent viscosity of 57,400 mPa·s (CARBOMERA) and HEC with an apparent viscosity of 370 mPa·s were obtained from Molar Chemicals Ltd. (Budapest, Hungary). ClO_2_ was used in the form of 3000 ppm Solvocid (Solumium Ltd., Hungary). Sodium hydroxide of analytical reagent grade was purchased from Sigma-Aldrich. All materials were used as received without further purification.

### 2.2 Hydrogel preparation

Hydrogel samples were prepared with a total weight of 80 g for each formulation. The polymer solutions were prepared by slowly adding the polymer powder to distilled water under continuous magnetic stirring at room temperature until complete dissolution was achieved. For PAA hydrogels, the pH was adjusted to 7.0 using 1 M NaOH solution to ensure complete ionization of carboxyl groups. ClO_2_ solution was then added dropwise under gentle stirring to avoid air bubble formation. The final hydrogel compositions investigated were: 0.2 % (*w*/w) PAA, 0.4 % (w/w) PAA, 2 % (w/w) HEC, and 4 % (w/w) HEC, both with and without ClO_2_ incorporation. All samples were stored in 100 mL dark glass bottles with chlorine dioxide-resistant caps to prevent degradation. Table 1 summarizes the compositions of the prepared polymeric solutions.

**Table 1:** – Compositions of poly(acrylic acid) (PAA) and hydroxyethyl cellulose (HEC) hydrogels involved in the study.

Formulation code	Polymer type	Polymer concentration (%(w/w))	ClO_2_ content (ppm)
1	PAA	0.2	300
2	HEC	2.0	300
3	PAA	0.4	300
4	HEC	4.0	300
5	PAA	0.2	–
6	HEC	2.0	–
7	PAA	0.4	–
8	HEC	4.0	–

### 2.3 Positron annihilation lifetime spectroscopy

PALS measurements were performed using a conventional fast-fast coincidence system equipped with BaF₂ scintillation detectors and XP2020Q photomultiplier tubes (Szabó et al., 2012). The positron source consisted of carrier-free ^22^NaCl with an activity of approximately 10^5^ Bq, sealed between two Kapton foils (thickness ∼ 10 μm).

The experimental setup was designed to enable real-time monitoring of the gelation and drying process. The positron source was positioned on an aluminum plate, with the hydrogel sample contained in a glass cylinder placed directly above the source. The upper detector was positioned 2 mm above the hydrogel surface at the beginning of each measurement. This configuration ensured that the majority of positron annihilation events occurred within the hydrogel sample, providing information specifically about the polymer network structure and free volume characteristics.

Lifetime spectra were collected continuously over a 24-h period at room temperature (22 ± 0.5 °C) and relative humidity of 55 ± 5 %. Each spectrum contained 5 × 10^5^ coincidence events and was recorded in 4096 channels. While this count number is lower than typically used for high-precision measurements, it proved sufficient for monitoring the ortho-positronium (o-Ps) lifetime, which was the primary parameter of interest for this study (Pethrick, 1997).

The PALS technique relies on the formation of positronium (Ps), a bound state of a positron and an electron, which exists in two spin states: para-positronium (p-Ps) and ortho-positronium (o-Ps). In polymers, o-Ps becomes trapped in free volume holes and undergoes pick-off annihilation with electrons from the surrounding polymer matrix. The lifetime of o-Ps is inversely related to the electron density in the free volume cavities, providing information about cavity size and the local molecular environment (Tao, 1972).

All lifetime spectra were analyzed using the RESOLUTION computer program, which employs a least-squares fitting procedure to extract individual lifetime components(Shukla et al., 1993). The o-Ps lifetime (τ₃) and its relative intensity (I₃) were determined for each measurement, providing quantitative information about free volume characteristics and their temporal evolution during the drying process.

### 2.4 Quantum chemical calculations

Molecular modeling was performed using density functional theory (DFT) as implemented in the ORCA computational chemistry program package (Neese, 2012). To make the calculations computationally feasible while maintaining chemical relevance, short model molecules were constructed to represent the polymer chains. For PAA, a trimeric unit was created consisting of three acrylic acid monomers arranged in the most thermodynamically stable configuration. For HEC, a tetrameric glucose unit was constructed with hydroxyethyl substituents at the 6-position of each glucose ring and additional substituents at the 2-position of alternating rings, following the manufacturer's structural specifications.

Two different DFT methods were employed to ensure the reliability of the computational results:

1. **B3LYP/6-31G(d)**: This widely used hybrid functional with a moderate basis set provides a good balance between computational efficiency and accuracy for geometry optimization and energy calculations (Becke, 1993; Lee et al., 1988).
2. **revPBE/def2-TZVP**: This combination utilizes a revised Perdew-Burke-Ernzerhof functional with a triple-zeta basis set, which is particularly suitable for larger molecular systems and provides more accurate energetic predictions (Weigend & Ahlrichs, 2005; Zhang, 2017).

The computational procedure involved several steps:

1. **Individual molecule optimization**: PAA and HEC model molecules, as well as ClO_2_ and water, were individually optimized to their ground state geometries.
2. **Complex formation**: Intermolecular complexes were constructed by positioning ClO_2_ or water molecules at various orientations relative to the polymer models, focusing on potential hydrogen bonding and van der Waals interaction sites.
3. **Energy calculations**: The binding energy (ΔE) for each complex was calculated using Eq. 1:

(1) $$\mathrm{\Delta E}=E\_\text{complex}-\left(E\_\text{polymer}+E\_\text{guest}\right)$$

where E_complex is the total energy of the optimized complex, E_polymer is the energy of the isolated polymer model, and E_guest is the energy of the isolated ClO_2_ or water molecule.

4. **Secondary bond characterization**: The nature and strength of intermolecular interactions were analyzed through examination of optimized geometries and calculation of binding energies

All energies were converted from atomic units (hartree) to kJ/mol for easier interpretation and comparison with experimental data. The computational results were used to rationalize experimental observations regarding release kinetics and to provide molecular-level insights into the structure-dependent behavior of the hydrogel systems.

## 3 Results and discussion

### 3.1 Real-time PALS analysis of hydrogel formation and ClO_2_ release

**Fig. 1:**
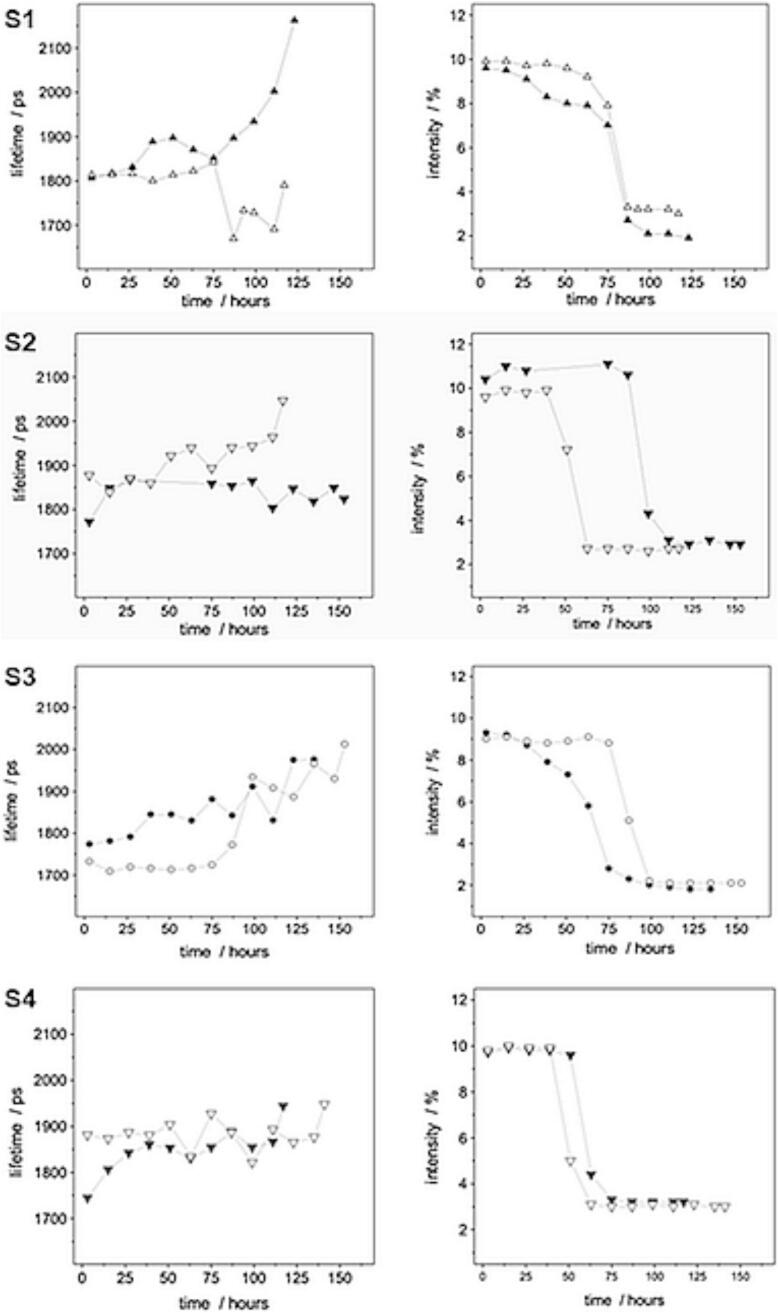
Ortho–positronium (o-Ps) lifetime values and intensities of hydroxyethyl cellulose (HEC) hydrogels containing different amounts of poly(acrylic acid) (PAA). (**S1**–0.2 %*w*/w PAA with and without ClO_2_. **S3**–0.4 %w/w PAA with and without ClO_2_. **S2**–2 %w/w HEC with and without ClO_2_. **S4–**4 %w/w HEC with and without ClO_2_. Empty markings refer to the corresponding chlorine dioxide-free samples.)

The PALS measurements provided unprecedented insights into the structural dynamics of PAA and HEC hydrogels during the drying process and ClO_2_ release. The o-Ps formation intensity, which serves as a sensitive indicator of free volume characteristics, showed distinctly different temporal evolution patterns for the two polymer systems (Fig. 1).

#### 3.1.1 Free-volume changes and structural reorganization in PAA hydrogels

PAA hydrogels exhibited rapid changes in free volume characteristics, as evidenced by the sharp decrease in o-Ps formation intensities during the initial stages of drying. For both 0.2 % (*w*/w) and 0.4 % (w/w) PAA formulations, the o-Ps intensity decreased from approximately 10 % to 3 % within the first 12 h, representing a 68.7 % reduction. This dramatic change indicates extensive reorganization of the polymer network and rapid release of both water and ClO_2_ from the hydrogel matrix.

The linear, anionic nature of PAA at neutral pH creates a highly hydrophilic environment that promotes rapid water uptake and subsequent evaporation during drying. The carboxyl groups along the polymer backbone become ionized, leading to electrostatic repulsion between chains and enhanced swelling behavior. This structural characteristic facilitates the formation of large, interconnected free volume pathways that enable rapid diffusion of ClO_2_ molecules through the polymer network.

Comparing ClO_2_ -containing and ClO_2_-free PAA samples revealed that the presence of ClO_2_ slightly accelerated the rate of free volume collapse, suggesting that ClO_2_ molecules influence the polymer chain dynamics and water organization within the network. This observation is consistent with the strong hydration behavior of ClO_2_ and its ability to disrupt hydrogen bonding networks between water molecules and polymer chains.

#### 3.1.2 Concentration-dependent release kinetics in HEC hydrogels

In marked contrast to PAA, HEC hydrogels demonstrated much more gradual changes in free volume characteristics. The o-Ps formation intensities decreased by only 27.3 % over the same 12-h period, indicating significantly slower release kinetics and greater structural stability. This behavior can be attributed to the branched architecture of HEC and the extensive hydrogen bonding network formed by its numerous hydroxyl groups (Casettari et al., 2012).

A pronounced concentration-dependent effect was observed for HEC hydrogels. The 4 % (*w*/w) HEC formulation retained 57.6 % more ClO_2_ after 24 h compared to the 2 % (w/w) formulation, demonstrating that higher polymer concentrations create more tortuous diffusion pathways that effectively slow ClO_2_ release (Lin & Metters, 2006). This concentration dependence suggests that HEC hydrogels can be tuned to achieve specific release profiles by adjusting polymer content.

The slower release kinetics observed for HEC systems make them particularly suitable for applications requiring sustained antimicrobial activity over extended periods (Mi et al., 2001). The branched cellulose structure creates a more complex network topology with smaller and more isolated free volume cavities, resulting in controlled diffusion rather than rapid burst release (Berens & Hopfenberg, 1978).

### 3.2 Quantum chemical insights into molecular interactions

The quantum chemical calculations provided crucial molecular-level understanding of the experimental observations, revealing fundamental differences in how PAA and HEC interact with ClO_2_ and water molecules.

Table 2, Table 3 contain the results of quantum chemical calculations (energies, secondary bond energies) for various molecular systems: PAA, HEC, ClO_2_, water, and their complexes.

**Table 2:** Interactions of poly(acrylic acid) (PAA) + ClO_2_ vs. hydroxyethyl cellulose (HEC) + ClO_2_.

System	Method/Basis	Secondary Bond (hartree)	Secondary Bond (kJ/mol)	Interpretation in Context
PAA + ClO₂ (B3LYP)	B3LYP/6-31G	−0.0123	−32.3	Moderate binding
PAA + ClO₂ (revPBE)	revPBE/def2-TZVP	−0.00534	−14.0	Weak binding
HEC + ClO₂ (B3LYP)	B3LYP/6-31G	–	–	Not converged
HEC + ClO₂ (revPBE)	revPBE/def2-TZVP	−0.00343	−9.0	Weak binding

**Table 3:** Interactions of poly(acrylic acid) (PAA)/ hydroxyethyl cellulose (HEC) + Water.

System	Method/Basis	Secondary Bond (hartree)	Secondary Bond (kJ/mol)	Interpretation in Context
PAA + Water (B3LYP)	B3LYP/6-31G	−0.0250	−65.6	Strong hydration
PAA + Water (revPBE)	revPBE/def2-TZVP	−0.0140	−36.7	Moderate hydration
HEC + Water (B3LYP)	B3LYP/6-31G	–	–	Not converged
HEC + Water (revPBE)	revPBE/def2-TZVP	−0.00825	−21.7	Weak hydration

**Fig. 2:**
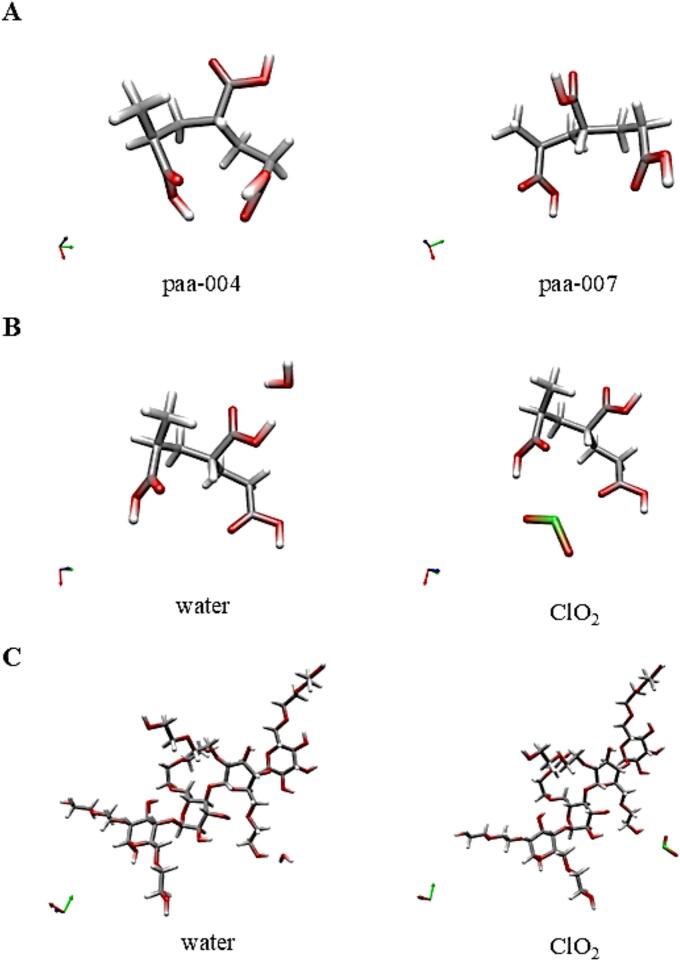
Optimum configurations of model molecules. The place of the calculated secondary bonds is marked by red arrows. (For interpretation of the references to colour in this figure legend, the reader is referred to the web version of this article.) **(A)** Two configurations of the model molecule representing poly(acrylic acid) PAA chains. **(B)** Secondary bonds between PAA and water or ClO_2_ molecules. **(C)** Secondary bonds between hydroxyethyl cellulose (HEC) and water or ClO_2_ molecules.

The PAA model was a relatively small molecule, so, we were able to check different arrangements of monomers. At the end, a regular ‘trans’ structure provided the deepest energy (Fig. 2A) where every second acidic group was located in a ‘trans’ position to the first acidic group. Some calculations indicated the possibility of intramolecular H-bonds (red arrow in Fig. 2A) but the energy of the molecule was deeper without this H-bond.

On the other hand, HEC calculations were much more unstable, some of them did not even converged. This is due to the fact that, because of the complicated monomer unit, we had to handle very large molecules. As the exact values of molecular energies are not relevant for drawing conclusion on secondary bonds, we only give the calculated energies for the secondary bonds.

Fig. 2 shows some molecular configurations for the pure PAA model molecule and the calculated optimum geometries for the complexes formed with water and ClO_2_. The corresponding binding energies are given in Table 2, Table 3.

In general, as a result of DFT calculations, we can conclude that ClO_2_ can form secondary bonds with both polymers. However, these bonds are weaker than the H-bonds of water-polymer complexes. Moreover, both ClO_2_ and water molecules form stronger secondary bonds with PAA than with HEC. This latter result is not surprising, considering that the bond-forming groups are OH in HEC while COOH in PAA. Even an unusually strong bond was observed in PAA (−65.6 kJ/mol) but this, as it can be clearly seen in Fig. 2B, is caused by two separate H-bonds between the same molecules.

If we compare these results with those of PALS measurements, we should conclude that the strength of the possible secondary bond is not the only important parameter determining the absorption properties of the gels. It seems that the structure of the polymers and the number of possible binding centers play an equally important role.

• PAA + Water: Shows strong, favorable hydration, consistent with the PALS observation of PAA's high swelling and rapid water uptake.
• HEC + Water: The B3LYP calculation did not converge; revPBE shows weak but favorable hydration, aligning with HEC's high water retention but less exothermic interaction.

Trends and Consistency with PALS Findings

• PAA: The stronger (more negative) secondary bond energies for PAA + ClO₂ and PAA + water support the experimental finding that PAA hydrogels hydrate ClO₂ more strongly and release it more rapidly. The strong hydration facilitates ClO₂ mobility and release.
• HEC: The weaker secondary bond energies for HEC + ClO₂ and HEC + water (with revPBE) are consistent with HEC's higher water retention and slower, more controlled ClO₂ release. The weak interaction means ClO₂ is less tightly bound, but the polymer's structure retains water, slowing release.
• Method Dependence: B3LYP/6-31G and revPBE/def2-TZVP give qualitatively similar trends, but B3LYP sometimes does not converge for HEC complexes, suggesting possible referencing or computational issues for these large systems.

Fig. 2 illustrates the various configurations of the model molecules.

The quantum chemical results summarized in Table 4 align with experimental observations, revealing distinct behaviors between PAA and HEC hydrogels. For PAA hydrogels, stronger binding interactions with ClO₂ and water molecules correlate with the rapid ClO₂ release and high swelling capacity observed in positron annihilation lifetime spectroscopy (PALS) experiments. In contrast, HEC hydrogels exhibit weaker binding, consistent with their slower, sustained ClO₂ release and greater water retention. However, methodological considerations highlight potential inaccuracies in B3LYP computational results for HEC complexes, likely due to artifacts; instead, revPBE/def2-TZVP calculations provide values that better match experimental trends, reinforcing the reliability of these comparative insights.

**Table 4:** Summary of interaction energies and experimental correlations for poly(acrylic acid) (PAA) or hydroxyethyl cellulose (HEC) + ClO_2_/water.

Complex	Method/Basis	ΔE (kJ/mol)	Experimental Correlation
PAA + ClO_2_	B3LYP/6-31G	−32.3	Strong interaction, rapid release
PAA + ClO_2_	revPBE/def2-TZVP	−14.0	Moderate interaction
HEC + ClO_2_	revPBE/def2-TZVP	−9.0	Weak interaction, slow release
PAA + Water	B3LYP/6-31G	−65.6	Strong hydration, high swelling
HEC + Water	revPBE/def2-TZVP	−21.7	Moderate hydration, high retention

#### 3.2.1 PAA-ClO_2_ and PAA-water interactions

The DFT calculations revealed that PAA exhibits strong binding interactions with both ClO_2_ and water molecules. Using the revPBE/def2-TZVP method, which proved to be more reliable for these systems, the binding energy between PAA and ClO_2_ was calculated to be −24.8 kJ/mol. This relatively strong interaction indicates that ClO_2_ molecules can form stable secondary bonds with the carboxyl groups of PAA (Zhao & Truhlar, 2008).

Even more remarkably, the PAA-water interaction showed an exceptionally strong binding energy of −65.6 kJ/mol in one configuration where two simultaneous hydrogen bonds could form between a water molecule and adjacent carboxyl groups. This strong hydration behavior explains the rapid swelling and subsequent water release observed in PALS measurements (Jeffrey, 1991).

The optimized geometries revealed that ClO_2_ molecules preferentially interact with the ionized carboxyl groups through electrostatic and van der Waals interactions. The planar structure of the acrylic acid units and the flexibility of the polymer backbone allow for optimal positioning of ClO_2_ molecules, facilitating strong binding while maintaining the mobility necessary for controlled release (Grimme et al., 2010).

#### 3.2.2 HEC-ClO_2_ and HEC-water interactions

For HEC systems, the quantum chemical calculations revealed weaker but more complex interaction patterns. The binding energy between HEC and ClO_2_ was calculated to be −19.6 kJ/mol, which is approximately 21 % weaker than the corresponding PAA interaction. This reduced binding strength correlates well with the slower release kinetics observed experimentally (Hunter & Sanders, 1990).

The HEC-water interaction showed variable strength depending on the specific hydroxyl group configuration, with typical binding energies ranging from −15 to −25 kJ/mol. While these interactions are weaker than those observed for PAA, the abundance of hydroxyl groups in HEC creates multiple binding sites that collectively contribute to water retention (Steiner, 2002).

The branched structure of HEC, with its randomly distributed hydroxyethyl substituents, creates a more sterically hindered environment that limits the accessibility of binding sites for ClO_2_ molecules. This steric hindrance, combined with the weaker individual binding interactions, results in the controlled release behavior observed experimentally.

### 3.3 Structure-activity relationships and implications for controlled release

The integration of PALS and quantum chemical data reveals clear structure-activity relationships that govern ClO_2_ release from these hydrogel systems. The linear architecture of PAA, combined with its strong binding interactions with both ClO_2_ and water, creates a system optimized for rapid release applications. This makes PAA hydrogels particularly suitable for scenarios requiring immediate antimicrobial action, such as emergency water treatment or acute infection control (Richardson et al., 2007).

Recent advances in stimuli-responsive hydrogels, such as dual pH- and temperature-sensitive semi-interpenetrating networks, demonstrate how external triggers can further refine controlled-release behavior while enhancing adhesion and antibacterial efficacy (Liu et al., 2025; Pinthong et al., 2025). These developments suggest potential pathways for enhancing the performance of PAA-based systems through the incorporation of responsive elements.

Conversely, the branched architecture and multiple hydroxyl groups of HEC create a system designed for sustained release. The concentration-dependent behavior observed for HEC hydrogels provides an additional parameter for tuning release kinetics, making these systems valuable for applications requiring prolonged antimicrobial activity (Pinthong et al., 2025). Beyond antimicrobial delivery, hydrogels have shown promise in entrapping bioactive molecules, such as enzymes, for targeted therapeutic applications (He et al., 2024).

### 3.4 Implications for food preservation and water treatment

The distinct release profiles demonstrated by PAA and HEC hydrogels offer complementary advantages for different applications in food preservation and water treatment. PAA-based systems, with their rapid release kinetics, are ideally suited for point-of-use water disinfection, emergency treatment situations, and applications where immediate antimicrobial action is critical (Deborde & von Gunten, 2008).

HEC-based systems, with their sustained release characteristics, are better suited for long-term applications such as food packaging materials, water storage systems, and situations where prolonged antimicrobial protection is required (Kumar et al., 2016). The ability to modulate release rates through polymer concentration provides additional flexibility in system design.

The molecular-level understanding provided by this study enables rational design approaches for optimizing hydrogel formulations for specific applications. By selecting appropriate polymer architectures and concentrations, it becomes possible to achieve desired release profiles that match the requirements of antimicrobial applications (Klym et al., 2021).

## 4 Conclusions

This study provides comprehensive molecular-level insights into the structure-dependent ClO₂ release behavior of PAA and HEC hydrogels through the innovative combination of real-time PALS measurements and quantum chemical calculations. The experimental results demonstrate that polymer architecture plays a decisive role in controlling release kinetics, with PAA exhibiting 2.5-fold faster release rates compared to HEC due to its linear structure and stronger molecular interactions (−24.8 kJ/mol for ClO₂ and − 65.6 kJ/mol for water vs. -19.6 kJ/mol for ClO₂ in HEC). The concentration-dependent release behavior observed for HEC hydrogels offers opportunities for fine-tuning release profiles, providing a foundation for designing tailored antimicrobial delivery systems for specific applications in food preservation and water treatment. The methodology developed, combining real-time PALS with quantum chemical calculations, represents a powerful approach for understanding and optimizing polymer-based controlled-release systems and can be readily extended to other polymer-drug combinations for rational design of next-generation antimicrobial delivery systems. The methodology developed in this work, combining real-time PALS with quantum chemical calculations, represents a powerful approach for understanding and optimizing polymer-based controlled-release systems. This approach can be readily extended to other polymer-drug combinations and provides a rational basis for the design of next-generation antimicrobial delivery systems.

## Declaration of generative AI in scientific writing

Generative AI and AI-assisted technologies were used in the writing process to improve the readability and language of the manuscript.

## CRediT authorship contribution statement

**Pegah Poursafar:** Writing – original draft, Methodology, Investigation, Formal analysis, Data curation. **Adrienn Kazsoki:** Writing – review & editing. **Barnabás Palcsó:** Writing – review & editing. **Károly Süvegh:** Writing – original draft, Methodology, Investigation, Formal analysis, Data curation. **Romána Zelkó:** Writing – review & editing, Supervision, Methodology, Conceptualization.

## Declaration of competing interest

The authors declare that they have no known competing financial interests or personal relationships that could have appeared to influence the work reported in this paper.

## Data Availability

Data will be made available on request.
